# Hemodynamic Factors Affecting the Suitability of the Donated Heart and Kidney for Transplantation

**Published:** 2013-11-01

**Authors:** B. Nozary Heshmati, F. Ahmadi, P. Azimi, N. Tirgar, F. Barzi, S. M. Gatmiri

**Affiliations:** 1 *Iranian Tissue Bank and Research Center, Tehran University of Medical Sciences, Tehran, Iran *; 2 *Nephrology Research Center, Tehran University of Medical Sciences, Tehran, Iran*; 3 *Shahid Beheshti University of Medical Sciences, Tehran, Iran*

**Keywords:** Hemodynamics, Suitability of heart and kidney, Brain death, Deamino arginine vasopressin, Desmopressin

## Abstract

Background: Due to the loss of autonomic nervous system, precise control of the hemodynamic status in dead brain potential donors presents a clinical dilemma. In these patients, due to head trauma and cerebral edema, fluids administration is restricted. Moreover, the decreased central venous pressure may put the viability of the organs at risk.

Objective: To investigate hemodynamic factors affecting the suitability of the donated heart and kidney for transplantation.

Methods: Data were retrospectively collected from the maintained databases of all dead-brain donors (DBDs) admitted to our organ procurement unit (OPU) ICU between 1999 and 2008. In this study, laboratory variables in addition to demographic data were collected. The time between donor entrance to the DBD ICU and organ procurement, vital signs, hourly urine output, amount of IV fluid administered, and the dosage of vasopressor and desmopressin were recorded. The end-point of the study was organ suitability for organ retrieval.

Results: A total of 132 dead brain donors were studied. The mean±SD age of the donors was 26.3±12.2 years. The main cause of brain death was multiple trauma (53%). The organ retrieval rate was 82.6% for the kidney, 59.8% for the liver, and 53% for the heart. 83 (63%) and 106 (80.3%) donors had suitable hearts and kidneys, respectively. 66 cases did not receive desmopressin (50.4%) at all. The mean±SD dose of desmopressin the donors received was 7±1 µg. There was a significant association between the suitability of these two organs for transplantation and the dosage of the administered desmopressin and volume of IV solution the donors received.

Conclusion: Fluid therapy and administration of desmopressin can improve the number and quality of retrieved organs from dead brain donors.

## INTRODUCTION

The demand for donor organs is increasing continuously. Iran is among the countries that try to increase the rate of dead-brain donors (DBDs). However, a potential DBD should be transferred to the ICU of organ procurement units (OPUs) by the coordinating team, for lack of technical expertise and human resources for managing the donor’s medical conditions. Brain death is associated with immunological and endothelial activation, cytokine and catecholamine storm which may provoke delayed graft function. The main responsibility of the management team is to maintain the arterial blood pressure and hemodynamic status of the donor to preserve as many organs as possible for retrieval. The balance between the least dose of vasopressors and optimal amount of volume therapy needs effective supervision and although there are various guidelines, the rate of hemodynamic and metabolic abnormalities in DBDs is still high. This issue is much more important when the management is separated in two ICUs of the hospital of origin and referral OPU [1-3].

Hemodynamic control in DBD is a serious clinical challenge not only due to the donor’s physiologic situations but also because of the involvement of a high number of specialties in decision making. The usual practice in patients with multiple trauma, before declaration of the brain death, is to restrict free fluid administration. Furthermore, administration of diuretics is sometimes inevitable for cerebral protection. Insufficient volume therapy, central venous hypotension and high-dose vasopressors may cause vital organs damage. Moreover, one strategy that may be helpful for preservation of one organ may not be useful for the other. For example, the high rate of lung recovery may be affected by the restrictive volume balance, which is in sharp contrast with what has been recommended for kidney transplantation [4].

For impaired hemodynamics in DBDs, the efficacy of different protocols to maintain the patient’s hemodynamics including fluid infusion, administration of vasopressor and desmopressin, is variable and may lead to changes in cardiac index, arterial blood pressure, heart rate and even organ recovery rate [6, 7]. Furthermore, the quick performance of the team is important. Ramjug, *et al*, showed that longer delays between donor brain death and cross-clamp time are associated with a higher-risk of mortality in cardiac transplant recipients. The mean±SD cross-clamp time in their study was 13.2±3.96 hours [5].

We conducted this study to determine the hemodynamic factors affecting the suitability of the donated heart and kidney for transplantation.

## PATIENTS AND METHODS

Data were retrospectively collected from the databases of all DBDs admitted to our OPU ICU between 1999 and 2008. In this study, BUN and creatinine, and demographic data were collected. The time between donor entrance to our DBD ICU and organ procurement, vital signs, 24-hour urine output before organ procurement, amount of IV fluid administered, and dosage of vasopressor and desmopressin were recorded. The end-point of the study was organ suitability for organ retrieval. Potential suitability for transplantation of kidneys was defined by the last serum creatinine level before the organ procurement, 24-hour urine output before retrieval and approval of a nephrologist. Potential suitability for heart transplantation was defined by the dose of vasopressor administered and the mean blood pressure in during the last 24 hours, history of cardiac arrest and duration of CPR, and approval of a cardiologist. 

This study was exempted from the assessment and approval by the University Ethics Committee due to its retrospective nature. Brain death was diagnosed according to the protocol practiced in Iran [8]. Statistical analyses were performed by SPSS^®^ for Windows^®^ ver 19.0 (SPSS, Inc., Chicago, IL, USA). Descriptive data at the time of admission and during the follow-up are presented as mean±SD. Continuous and categorical variables were compared between the two groups using the *Student’s t *test for independent samples, and χ^2^ test, respectively. A p value <0.05 from 2-sided tests was considered statistically significant. 

## RESULTS

This study included 132 cases of DBDs. The male to female ratio was 1.8 (85/47); they had a mean±SD age of 26.3±12.2 (range: 7–60) years. Two donors (1.5%) had a history of hypertension. The main causes of brain death were head trauma due to traffic collisions (53%), falling down (13.6%), and cerebrovascular accidents (11.4%). The donors’ characteristics with respect to their hemodynamics and the dose of administered inotrope are summarized in Table 1. There was no significant correlation between the cause of brain death and suitability of the harvested organs for transplantation.

**Table 1:** Characteristics of studied DBDs. Values are presented as mean±SD.

Variable	At the time of entrance to ICU	12 hours after admission	before organ recovery
MAP* (mm Hg)	8.28±1.8	8.61±1.7	8.78±2.01
CVP (cm H_2_O)	8.12±11.1	7.28±4.4	10.5±9.8
Dopamine dose (µg/kg/min)	5.5±3.7	5.0±2.9	4.7±3.1
Heart rate (beats/minute)	90.9±19.5	87.8±21.2	90.8±23.3

* Mean arterial blood pressure

The mean±SD time from the onset of coma to the clinical diagnosis of brain death was 61.7±55.7 hours; the duration from the entrance to the OPU ICU until organ procurement was 20.1±5.5 hours. Thirty-five donors (26.5%) received only crystalloid IV solutions, while 55 (41.7%) received dextrose/crystalloid IV fluids. Th donors received a mean±SD of 52.7±25.8 (range: 8–98) mL/h fluid. Thirty-three donors (25%) did not receive bicarbonate in their volume therapy.

Seven (5.3%) donors needed packed RBC, 13 (9.8%) received fresh frozen plasma (FFP), 15 (11.4%) received both packed RBCs and FFP, and two received platelets. Twenty-two (16.7%) DBDs experienced cardiac arrest; the mean±SD duration of CPR was 2.1±0.65 (range: 0–8) minutes.

Inotropic agents used included dopamine in 95 (72%), and dobutamine and epinephrine each in one (0.8%).No record was found in 27 donors (4.20%). Sixty-six cases did not receive desmopressin (50.4%) at all. The mean±SD dose of desmopressin administered was 7±1 µg; the urine output 12 h before organ recovery was 217.1±153.9 (range: 11.5–942) mL/h. Seventy-four cases (70.5%) had polyuria (urine output >125 mL/h).

The utilization rates were 82.6% for the kidneys, 59.8% for the liver, 53% for the heart, 3% for the lungs and 0.8% for the pancreas. Seventy (53%) hearts were recovered. Potential suitability for transplantation of hearts in each donor was optimal in 83 (62.9%), moderate in 33 cases (25%), and unsatisfactory in 11 (8.3%) cases; no reliable data were available for the analysis in five cases (3.8%). The reasons for non-retrieval of suitable heart from DBDs were lack of supportive services to prepare the operating room, transplant team or potential recipients. Potential suitability for transplantation of hearts was not associated with age, sex, cause of brain death, changes in mean arterial blood pressure and heart rhythm, fluid intake in 24 hours before the surgery and dose of the administered desopressin in 24 hours prior to the organ harvesting procedure. The uitability was associated with administration of desmopressin (p=0.009) and type of the IV fluid received (p=0.03).

Kidneys were recovered from 109 DBDs (82.6%). Potential suitability for transplantation of kidneys in each donor was optimal in 106 cases (80.3%), moderate in 10 (7.6%), and unsatisfactory in six cases (4.5%); no reliable data were available for the analysis in 10 cases (7.6%). Regarding renal function, we found that the mean serum level of creatinine was 1.2 (range: 0.5–3.2) mg/dL at the time of admission and 1.1 (0.4–4.8) mg/dL prior to organ procurement.

There was no significant association between kidney suitability for transplantation and age, gender, cause of brain death, chnges in blood pressure, heart rate and dose of the administered vasopressor (in primary hospital, at ICU entrance, and prior to organ procurement). However, we found a significant correlation between successful kidney recovery with the volume of the administered IV fluid (p=0.006), the type of IV fluid which could provide a better hemodynamic status (p<0.001), and administration of desmopressin (p=0.002) within 24 hours prior to organ recovery and its dose (p=0.008).

## DISCUSSION

The main purpose of potential DBD care is to stabilizing and controlling the hemodynamic status of DBD to protect organs as best as possible. Two findings in our study require attention. The first was the young age of our donors, which was similar to other studies in Iran and other developing countries; this is in sharp contrast with many western countries discussed thoroughly elsewhere [2, 8]. The second was the cause of brain death. We did not find any association between the causes of brain death and organ suitability. The main causes of brain death in our series were traffic collisions and falling down. However, Lee, *et al*, in their study on 299 cases, found two main factors predicting the higher risk of kidney graft failure: cerebrovascular accident (CVA) as the cause of brain death and history of hypertension which is in contrast with our study. In their study, 43% (128/299) of the donors had the two mentioned criteria—CVA as the cause of death or history of hypertension [9]. It seems that the difference age distribution between the two studies and causes of death would be important enough to lead to obtaining different results. 

The mean±SD duration between arriving at the OPU ICU and organ procurement was 20.1±5.5 hours—more than some other studies [5]. The transfer of the potential DBD from ICU of hospital of origin to the referred OPU ICU may prolong the unwanted pathologic processes in DBD.

The next issue, which is believed by all researchers involved in DBD management, is the administration of desmopressin. Diabetes insipidus may cause a rapid reduction in intravascular volume, hypotension and abnormal serum electrolytes. Therefore, the main steps in DBD management are volume therapy and administration of desmopressin. Increasing the intravascular volume with colloids or crystalloids is required in DBD management which is routinely adjusted by CVP [8, 10, 11]. The incidence of diabetes insipidus in DBDs is 30%–90% [12]; it was seen in nearly 50% of our cases. Kazemeyni, *et al*, in their study on 57 kidney recipients from DBDs, found that the serum creatinine level on the 7th day and before discharging from the hospital (after transplantation) correlated significantly with the donor’s urine volume (r = 0.329, p=0.04), and serum sodium level (r = 0.316, p=0.02), respectively [12]. Plurad, *et al*, by using the Organ Procurement and Transplantation Network database, studied 10,431 donors and found that those who received desmopressin (n=7873, 75.5%) had a significantly higher yield of organ procurement than those who did not (50.5% *vs* 35.6%, p<0.001) [10]. Our study also showed a positive correlation between organ suitability for transplantation and administration of desmopressin and crystalloid solutions. 

In the present study, we did not find any correlation between suitability of heart and kidney for transplantation and the dose of the administered inotropes, which may be due to the younger age and more stable hemodynamic state of the donors studied. The main variable that correlated with a higher rate of organ suitability was higher fluid therapy and the administration of inotropic agents. 

As administration of desmopressin and IV fluid has a positive impact on the suitability of organs for transplantation, it seems necessary to follow the recipients to further confirm these results.
